# Photon-Counting CT-Angiography to Assess Intracranial Stents and Flow Diverters in Comparison to Digital Subtraction Angiography

**DOI:** 10.1007/s00062-025-01519-2

**Published:** 2025-05-09

**Authors:** Frederic De Beukelaer, Mohammed El Halal, Sophie De Beukelaer, Laura L. Wuyts, Martin Wiesmann, Hani Ridwan, Charlotte S. Weyland

**Affiliations:** 1 Department of Diagnostic and Interventional Neuroradiology, University Hospital RWTH Aachen, Aachen, Germany; 2 Department of Neurology, Inselspital, University hospital Bern, Bern, Switzerland; 3 Department of Radiology, AZ Sint-Lucas, Ghent, Belgium

**Keywords:** Cerebral angiography, Cerebral arterial diseases, Computed tomography angiography, Intracranial aneurysm, Stent, Tomography, X‑ray computed

## Abstract

**Purpose:**

Photon-Counting Detector CT is characterized by enhanced image post-processing capabilities.

The diagnostic accuracy of PCD-CT angiography (PCD-CTA) in assessing intracranial stents (ICS) and flow diverters (FD) has yet to be compared with digital subtraction angiography (DSA).

**Methods:**

Retrospective analysis of all consecutive patients who underwent ICS or FD implantation between April 2023 and May 2024. Polyenergetic images, along with virtual monoenergetic imaging (VMI), pure lumen (PL) and iodine (IOD) reconstructions were assessed by three readers using a 5-point Likert scale and defined regions of interest (ROIs). A blinded analysis was performed to identify relevant lumen reduction. The diagnostic accuracy of PCD-CTA was compared to DSA by calculating the area under the receiver operating characteristic curve.

**Results:**

A total of 18 patients (mean age 59 ± 13 years; 14 women) with 14 implanted ICS and 10 FD were analyzed. Across all pairwise comparisons, pooled VMI reconstructions demonstrated higher ratings and signal-to-noise ratios compared to IOD, PL and UHR reconstructions (*p* < 0.001 for all comparisons). In the pooled assessment of DSA of in-stent vessel lumen 18 (11%) of the 162 vessel segments and 6 (33%) of the 18 patients presented relevant narrowing of the in-stent vessel lumen. The sensitivity of PCD-CTA for detecting stenosis was 100% (18 of 18 in-stent vessel segments), while specificity was 89% (128/144 in-stent vessel segments). All readers reported a 100% negative predictive value (128/128 in-stent vessel segments).

**Conclusion:**

Photon-Counting Detector CT might provide a reliable assessment of intracranial vessels following stent or flow diverter implantation comparable to DSA in many cases.

**Supplementary Information:**

The online version of this article (10.1007/s00062-025-01519-2) contains supplementary material, which is available to authorized users.

## Key Points

- Intracranial stents and flow diverter implantation may result in stenosis. Digital subtraction angiography (DSA) is a commonly used modality for follow-up.
- All readers observed a 100% negative predictive value using spectral reconstructions for detecting stenosis. Virtual monoenergetic imaging and iodine reconstructions provided higher signal to noise and contrast to noise ratios.
- Photon-Counting-CT-Angiography may reduce the need for DSA evaluations for patients after treatment of intracranial stenosis or aneurysms.

## Introduction

Intracranial stents (ICS) and flow diverters (FD) are employed to address symptomatic atherosclerotic disease (via ICS) or wide-neck intracranial aneurysms (through stent-assisted coil embolization or FD implantation). A significant complication associated with both device types is in-stent stenosis, which typically develops during the intimal coating phase within six months after implantation [1].

Additionally, flow diverter implantation can result in stent-end stenosis, characterized by focal narrowing, commonly referred to as “fish-mouthing” [2, 3].

Imaging protocols for follow-up to assess in-stent stenosis vary between hospitals. While MRI angiography and CT angiography can be affected by device-related artifacts, digital subtraction angiography (DSA) remains the gold standard for this purpose [4]. Flat-panel CT is another option for follow-up imaging, but not further addressed in this study.

The main challenges for the use of CT angiography (CTA) for follow-up imaging are the so-called blooming artifacts that lead to artificial lumen narrowing (ALN). Strategies to reduce blooming artifacts in CTA were higher resolutions scans and dual energy scans with subtraction and deep learning post-processing approaches with only partial reduction of artifacts. Thus, diagnostic subtraction angiography is still key to assess stent deformation, in stent thrombosis or stenosis due to its high resolution and its insusceptibility to metal artifacts [5, 6].

Photon-Counting detector CT angiography (PCD-CTA) is a new imaging modality, that offers high spatial resolution with phantom studies indicating its potential for improved stent assessment with reduced artifacts [7]. In comparison to the conventional Electron-Integrating Detector CT (EID-CT), Photon Counting CT measures each photon directly as electric signal. PCD-CTA allows for a higher image spatial resolution limit of 0.07 × 0.07 mm2 to 0.28 × 0.28 mm2 (interesting for example for evaluation of aneurysms, the inner ear or lung parenchyma). This is also possible because PCD-CT does not have separate scintillator elements and septa and can therefore be manufactured with smaller detector elements. Additionally, PCD-CT acquires spectral data, that can visualize material composition as for example iodine concentration. Spectral data allow for virtual monochromatic image reconstructoins. These spectral reconstructions should be adapted for imaging to different materials [8].

In vitro and in vivo studies suggest that the optimal threshold to identify iodine is 40 kilo electron Volt (keV) for monoenergetic reconstructions [9, 10].

Cardiovascular angiographies have identified higher kernels as better to evaluate coronary arteries: The vessel lumen of coronary arteries after stent implantation was best evaluated at a sharper reconstruction kernel (Body vascular kernel (Bv) 60 and 72) [11].

Virtual monoenergetic reconstructions with sharp kernels and low keV levels have been described as superior in assessing in-stent vessel lumen of intracranial stents and flow diverters [12].

To our knowledge, the assessment of ICS and FD has not yet been performed in vivo using PCD-CTA in comparison to DSA.

This study aims to evaluate the diagnostic accuracy of PCD-CTA for the assessment of intracranial stents and flow diverters compared to DSA as reference standard.

## Methods

The local ethics committee (Medical Faculty, University Hospital RWTH Aachen) approved the study and patient consent was waived. The research adhered to the STROBE criteria [13].

This analysis is a single-center, retrospective study of all consecutive patients treated for intracranial atherosclerotic disease with symptomatic, high-grade stenosis or wide-necked aneurysms using ICS or FD from April 2023 to May 2024. In daily routine, flow divertes for aneurysm treatment are approached in adherence to the recommendatios of Fiehler et al. [2].

The CT-Angiography was part of the clinical routine (assessment of stent deployment after intervention). All participants underwent imaging with a photon-counting CT scanner (NAEOTOM Alpha, software version syngo CT VB10; Siemens Healthineers, Erlangen, Germany) in ultra-high resolution (UHR) mode. The imaging protocol included contrast-enhanced PCD-CTA, centered on the implanted device and initiated via bolus tracking. Detailed scanning parameters are provided in the supplementary materials.

### Subjective Evaluation of Image Quality

Three radiologists with 10 (FdB) and 11 (LW, CW) years of experience independently evaluated all DSA and MRI images (workstation: syngo.via, version VB10A, Siemens Healthineers) while remaining blinded to clinical data.

The assessment focused on the visibility of the in-stent lumen at the proximal and distal ends and at the region with the minimal luminal diameter, using a 5-point Likert scale ranging from 1 (“non-diagnostic”) to 5 (“excellent”).

### Objective Evaluation of Image Quality

Contrast-to-noise ratios (CNR) and signal-to-noise ratios (SNR) were calculated and can be found in the online supplementary materials (Table S1). Two radiologists (blinded for review) independently placed ROIs to evaluate image quality. Five manually drawn ROIs, equal in diameter, were analyzed at the parent vessel, at the stent end, at the narrowest part of the stent, in the air rostral to the calvarium, and in the superficial temporal muscle. Additionally, measurements of vessel diameter were obtained pre-stent, post-stent and in the narrowest sections of the stent.

Hounsfield units were averaged from the ROIs, with exclusions for stent struts, vessel walls, and plaques were avoided. SNR and CNR were computed as follows:

$$\mathrm{SNR}=\text{signal}_{\text{artery}}/\text{standard deviation}_{\text{artery}}$$

$$\mathrm{CNR}=(\text{signal}_{\text{artery}}-\text{signal}_{\text{muscle}})/\text{standard deviation}_{\mathrm{air}}$$

### Assessment of In-stent Stenosis in UHR PCD-CTA and DSA

Three readers (FdB, LW and CW) independently quantified stenosis, remaining blinded to the DSA results. The stenosis diameter was defined, with significant lumen narrowing identified as ≥ 50% compared to the healthy proximal parent vessel diameter. Non-diagnostic segments due to poor image quality were considered as potential stenosis.

### DSA as Reference Standard Measurement

DSA was conducted with standard techniques by board-certified interventional neuroradiologists, with access gained via the radial or common femoral artery. At least two projections of the in-stent vessel lumen were obtained. A dedicated study read was performed independently by three readers (FdB, LW, CW), who remained blind to the DSA reports.

### Assessment of Radiation Dose

Radiation dose parameters, including dose length product (DLP) and dose area product (DAP), were documented from CT and DSA reports.

### Statistics

Statistical analysis was performed using IBM SPSS Statistics software (version 28.0). Depending on the distribution pattern, which was analyzed using a one-sample Shapiro Wilk test, quantitative variables were expressed as mean and standard deviation (range) or median and interquartile ranges (IQR).

Inter observer agreement was evaluated with Kendall’s coefficient of concordance (W) for both image quality and relevant in-stent stenosis (≥ 50% for DSA and PCD-CTA) and interpreted as follows: ≤ 0.20 none, 0.21–0.40 fair, 0.41–0.60 as moderate, 0.61–0.80 as substantial, and ≥ 0.81 as very strong agreement.

The scores were pooled to compare reconstructions (IOD, PL, UHR, VMI) via pairwise comparisons using Friedman’s test at three stent sites: proximal stent end, narrowest part of the stent, and distal stent end.

SNR and CNR were compared across the parent vessel, proximal stent end and narrowest part of the stent.

Krystal-Wallis-test was used to compare 1) patients with minimal in stent vessel lumen diameter of below and above 2 mm, 2) patients with stents and patients with flow diverter and 3) between patients with and without additional embolization devices (WEB-device, coil, clip). To assess the diagnostic performance of UHR PCD-CTA relative to DSA, pooled reader assessments across three in-stent lumen sites among the 18 patients were subjected to receiver operating characteristic curve analysis to compute the area under the curve (AUC). Nonparametric distribution assumptions were made for standard error approximation.

Sensitivity, specificity, positive predictive value and negative predictive value were computed for stenosis ≥ 50% compared to the healthy proximal parent vessel diameter at the in-stent segment level.

Results for all diagnostic accuracy tests were expressed with a 95% confidence interval (CI). A *p*-value of < 0.05 was considered statistically significant. Visualization of results was conducted using ggplot2 and Likert packages within R Software (The R Project for Statistical Computing, r‑project.org) [14, 15].

## Results

Patient characteristics are summarized in Table 1. A total of 18 patients (mean age 59 ± 13 years; range 37–84; 14 women) with 14 implanted intracranial stents and 10 flow diverters were analyzed. The minimal in-stent vessel lumen diameter was 1.9 ± 1.4 mm (1–3.7). Five patients (28%) received more than one device (ICS or FD), and 3 patients (17%) had additional embolization material (coils or WEB-devices).

**Table 1:** Patient characteristics

Characteristics	Number
Age (years) *	58.7 ± 13.1
*Sex (female/male)*	14/4
*Intracranial stenosis/Aneurysms*	6/13
*Number of flow diverters/Stents Implantate in the given location *	
ICA	8/1
ACA	0/2
MCA	0/4
Basilaris	0/2
PCA	1/0
Vert	1/0
*Flow diverter*	
Acandis Derivo	3
p64 MW HPC	6
p48 HPC	1
*Stent*	
CREDO	2
Acclino	4
Coroflex neo	1
Neuroform Atlas	1
*Additional embolization material*	
Clips	1
Coils	1
Clips and Coils	1
*Mean*_*minimal*_ in stent diameter*	1.9 ± 1.14
DLP (mGycm)*	67.7 ± 19.2
*DAP (µGym*^*2*^)*	2322 ± 340

Abbreviations: *ACA* anterior cerebral artery, *Basilaris* Basilar artery, *DAP* Dose-area product, *DLP* Dose-length product, *HPC* hydrophilic polymer coating, *ICA* internal carotid artery, *MCA* medial cerebral artery, *mGycm* Milligray centimeter, *mGycm*^*2*^ Milligray square centimeter, *N: PCA* Posterior cerebral artery, *Vert* vertebral artery

Data are numbers (*n*); n/N and if marked with an * Data are means ± standard deviations

No relevant in-stent vessel lumen stenosis was observed in patients with flow diverters; however, 4 out of 10 (40%) presented with fish-mouthing greater than > 50%.

**Fig. 1:**
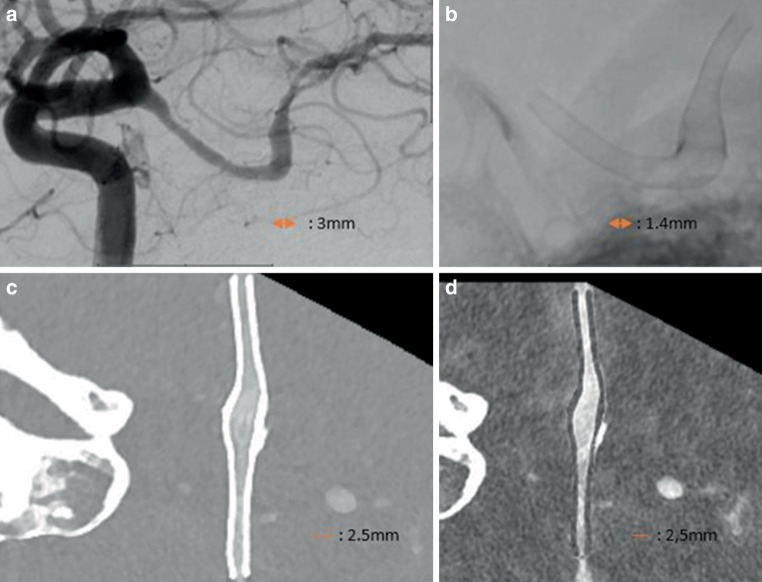
Two overlapping flow diverters in the right posterior communicating artery for the treatment of a fusiform aneurysm: **a** DSA lateral image, **b** blank image, radial reformations of the flow diverter of a VMI reconstruction **c** and iodine reconstruction **d** . Abbreviations: *DSA* Digital subtraction angiography, *PCD-CTA* Photon-Counting-Detector-CTA. *VMI* Virtual Monoenergetic Images

**Fig. 2:**
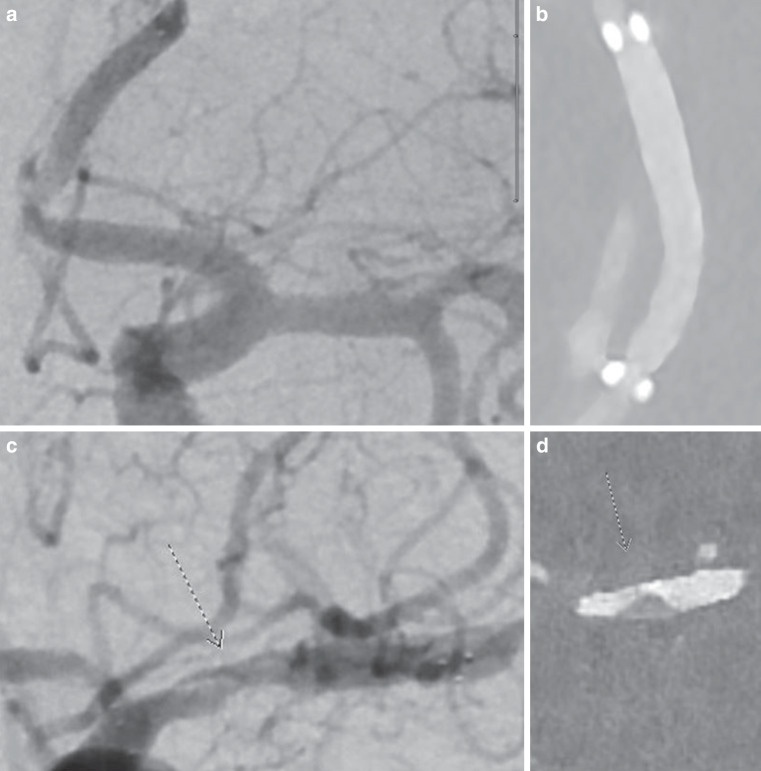
The p. a. image of the DSA (**a**) and the curved VMI reconstruction (**b**) show a stent in the A2 portion of the left anterior cerebral artery. The p. a. image of the DSA (**c**) and the VMI reconstruction (**c**) show a stent in the left middle cerebral artery with in-stent stenosis. Abbreviations: *Bv* Body vascular kernel, *DSA* Digital subtraction angiography, *VMI* Virtual Monoenergetic Images

Figure 1 shows DSA and PCD-CTA of a flow diverter the right posterior communicating artery and Fig. 2 shows examples of intracranial stents with in-stent stenosis and artificial lumen narrowing.

### Subjective and Objective Evaluation of Image Quality

In a cohort of 18 patients, 162 in-stent vessel segments were evaluated using four reconstruction methods: iodine density (IOD), pure lumen (PL), ultra-high resolution (UHR), and virtual monoenergetic imaging (VMI). Non-diagnostic ratings (Likert score 1) were observed in 9 vessel segments (5.3%) for IOD, 73 segments (43%) for PL, 29 segments (17%) for UHR and 8 segments (5%) for VMI.

Most segments were assessed as having good image quality (62 segments for IOD (36%), 22 for PL (13%), 49 for UHR (28%), and 49 for VMI (29%)) or excellent image quality (66 segments for IOD (39%), 15 for PL (9%), 48 for UHR (28%), and 104 for VMI (61%)).

In a pairwise comparison, VMI reconstructions were rated higher than IOD, PL, and UHR reconstructions (*p* < 0.001, *p* < 0.001, *p* = 0.001, respectively).

**Fig. 3:**
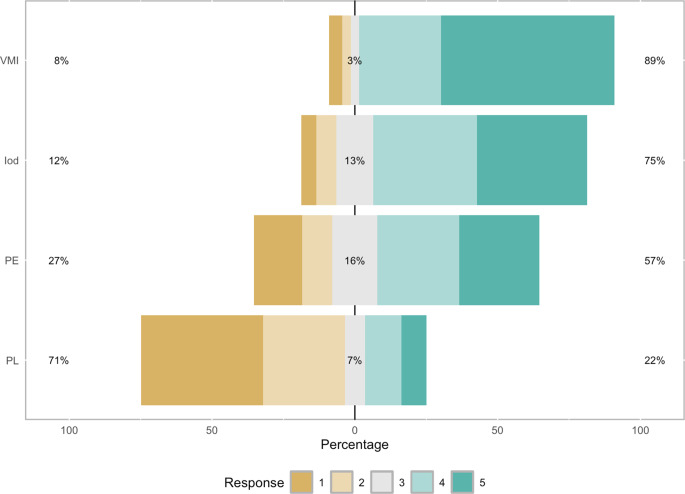
Qualitative image quality scores according to different spectral reconstructions, Iod, VMI, PL and PE. The stacked bar charts show pooled percentages of three raters. Interpretation of scores: 5 = excellent image quality, 4 = good image quality, 3 = acceptable image quality, 2 = barely satisfactory image quality, 1 = unacceptable image quality. *Iod* Iodine, *PE* polyenergetic, *VMI* virtual monoenergetic imaging, *PL* pure lumen)

Figure 3 displays Likert values of the different reconstructions.

The mean overall signal-to-noise-ratio (SNR) was 8.4 ± 3.9 (range 0.4–18.6) for iodine reconstructions (IOD), 5.7 (interquartile range [IQR] = 12) for pure lumen reconstructions (PL), 6.1 (IQR = 4) for UHR-reconstructions and 14 (IQR = 9.9) for virtual monoenergetic reconstructions (VMI). The mean overall CNR was 220.1 (IQR = 80.2) for IOD, 22.2 (IQR = 20) for PL, 9.6 (IQR = 5.9) for UHR and 20.7 (IQR = 11.9) for VMI.

**Fig. 4:**
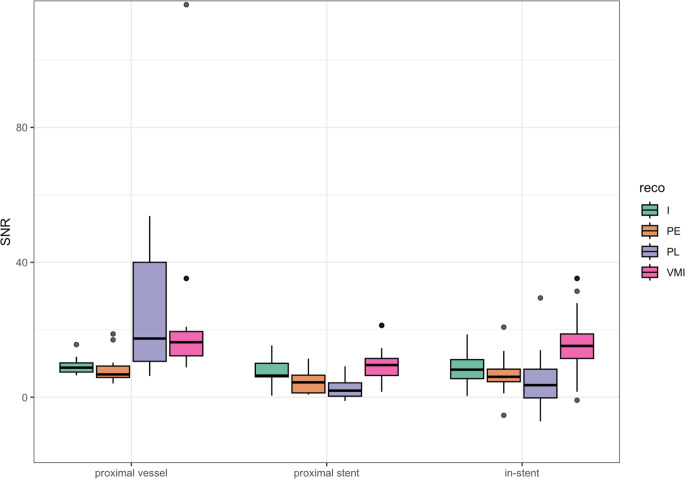
Box plot Distribution of Signal-to-Noise Ratio (SNR) in Photo-Counting-Detector Computed Tomography for different spectral reconstructions at three different sites. Iodine (IOD) reconstructions are coded in green, polyenergetic (PE) reconstructions in orange, pure lumen (PL) reconstructions in violet and virtual monoenergetic imaging (VMI) reconstructions in pink

VMI reconstructions provided higher SNR in visualizing in-stent vessel lumen compared to UHR, PL and IOD reconstructions (all *p* < 0.001) while IOD provided higher CNR compared to UHR, PL and VMI (all *p* < 0.001). SNR of the different reconstructions are visualized in Fig. 4.

The in-stent vessel diameter significantly affected the SNR of the proximal in-stent vessel lumen for all reconstructions except VMI (*p* = 0.10). Specifically, IOD (*p* = 0.02), PL (*p* = 0.14) and UHR (*p* = 0.04) reconstructions were influenced. The in-stent vessel diameter affected the CNR of the proximal in-stent vessel lumen only in PL reconstructions (*p* = 0.01), with no significant effects observed in iodine (*p* = 0.57), UHR (*p* = 0.374) and VMI (*p* = 0.51) reconstructions.

Lumen visibility was compromised by artifacts from stent markers across all reconstructions, particularly in vessels with small luminal diameters (all *p* < 0.001 for iodine, PL, UHR and VMI-reconstructions). Although flow diverter are braided stents, luminal assessment was better at the ends compared to stent body, with stent markers being a major source of artifacts (*p* = 0.01).

All spectral reconstructions and UHR were adversely affected by coils located near the in-stent vessel lumen (IOD-, PL-, UHR-, VMI-reconstructions: *p* = 0.005, *p* < 0.001, *p* < 0.001, *p* < 0.001 respectively).

### Diagnostic Performance of PCD-CTA Compared with DSA

In the pooled assessment of DSA of in-stent vessel lumen, 18 (10.5%) of the 162 vessel segments and 6 (32%) of the 18 patients presented with relevant narrowing of the in-stent vessel lumen.

Compared to DSA as gold standard, the overall sensitivity of PCD-CT, considering all spectral and UHR reconstructions for detecting stenosis, was 100% (18 of 18) and 89% (128/144), respectively. Excluding patients with artifacts due to additional coiling (3/18), specificity reached 94.1% (111/118).

The combined assessment of all spectral reconstructions for evaluation of the flow diverter lumen resulted in a positive predictive value of 90% (9/10) and a negative predictive value of 100% (128/128).

The area under the curve (AUC) was 0.94 (CI 0.911–l0.978). Illustrative Figure is provided in the electronic supplemental data.

Inter observer agreement was substantial for image quality (κ = 0.76, *p* < 0.001) and excellent for diagnosis of in-stent stenosis (κ = 0.92, *p* < 0.001).

### Assessment of Radiation Dose

The mean dose-length product for the CT scans and dose-area products for the DSA were as follows: PCD-CTA 67.7 ± 19.2 mGycm and DSA 2322 ± 340. µGycm^2^.

## Discussion

This study provides an in vivo evaluation of Photon-counting detector CT angiography (PCD-CTA) compared to DSA in assessing in-stent stenosis of flow diverters and intracranial stents.

When comparing spectral reconstructions of PCD-CTA, virtual monoenergetic reconstructions were rated significantly higher and showed higher signal-to-noise ratios than iodine-, pure lumen- and ultra-high resolution polyenergetic reconstructions (*p* < 0.001, *p* < 0.001, *p* = 0.001, respectively). The overall sensitivity of PCD-CTA with all spectral and ultra-high-resolution reconstructions for detecting stenosis was 100% (18/18 in-stent vessel segments) while its specificity was 90% (128/144 in-stent vessel segments). Excluding patients with major artifacts due to additional coiling (3/18 patients), specificity increased to 94.1% (111/118 in-stent vessel segments). All readers observed a 100% (128/128 in-stent vessel segments) negative predictive value.

These results align with findings in cardiovascular imaging. PCD-CTA for assessing coronary artery disease and stents in coronary arteries has demonstrated with high negative predictive values (100%) and a sensitivity and specificity of 100% and 92.3% for in-stent stenosis. However spectral reconstructions were not reported in those studies [11, 16–18].

Spectral image reconstruction methods allowed sufficient vessel lumen visualization even in vessels with diameters smaller than 2 mm, regardless of the device (flow diverter or intracranial stent). The potential of spectral image reconstruction has been published in in vitro studies for in-stent vessels, showing improved SNR with monoenergetic reconstructions compared to conventional reconstructions [7, 17].

To our knowledge, there are currently no existing in vivo studies on intracranial materials for comparing the feasibility of PCD-CTA and DSA as diagnostic follow-up. If these results are reproducible in a larger patient cohort, Photon-Counting-CT-Angiography may reduce the need for DSA evaluations for patients after treatment of intracranial stenosis or aneurysms.

Potential benefits for patients are a reduction in periinterventional risk and radiation exposure, as well as decreased costs for the healthcare system [19].

### Limitations

Our results lack generalizability concerning the following points:

- The patient cohort comprised only a limited number of intracranial stents and flow diverters overall as well as a limited number of patients with in-stent-stenosis and fish-mouthing of flow diverters. PCD-CTA might be of limited diagnostic qualities in braided stents (flow diverters) implanted in very small vessels (< 2 mm diameter). A scenario we did not study in this study cohort.
- As in other studies, stenosis was defined as more than 50% of lumen diameter proximal to the stent, but this is not an expression of hemodynamic or clinical relevance. Weather PCD-CTA can sufficiently differentiate between a moderate and a high-grade stenosis is still to be investigated.
- Although differences were found between the stent markers, the small number of stents analyzed does not allow for a comparative statement regarding in-stent lumen visibility between different stent and flow diverter products.
- In the vicinity of coils or clips, no reconstruction technique was able to overcome the consecutive metal artifacts. In these circumstances, DSA remained unchallenged.

Future studies on the diagnostic accuracy of photon-counting CT should include spectral reconstructions and a larger patient cohort, ideally with a larger variety of stents and flow diverters.

Additional to the higher spatial resolution, we consider spectral reconstructions in daily routine as possibility for advancing image quality.

## Conclusion

Compared to DSA, Photon-Counting Detector CT-Angiography can potentially provide a comparable assessment of intracranial vessels after stent or flow diverter implantation in the absence of additional materials like coils. Virtual monoenergetic imaging is pivotal for intracranial stent assessment compared to other possible spectral reconstructions of Photon-Counting CT-Angiography. Further studies are needed to define the diagnostic possibilities and diagnostic short-comings of PCD-CTA by assessing larger cohorts with different stents and flow diverters as well as stent implantations in very small intracranial vessels.

## Supplementary Information

Figure S: Receiver operating characteristic (ROC) curves of diagnostic accuracy of Photon-Counting Detector CTA compared to DSA

Table S1–S3

Scanning protocol

## Data Availability

Data and material are available from the corresponding author upon reasonable request.
