# Supplementary material for: Photon-Counting CT-Angiography to Assess Intracranial Stents and Flow Diverters in Comparison to Digital Subtraction Angiography
Source: Clin Neuroradiol. 2025 May 9;35(4):669–77. doi: 10.1007/s00062-025-01519-2 (PMC12552290; doi:10.1007/s00062-025-01519-2)
Supplement: Supplementary file 1 — Figure S: Receiver operating characteristic (ROC) curves of diagnostic accuracy of Photon-Counting Detector CTA compared to DSA [file 62_2025_1519_MOESM1_ESM.docx]

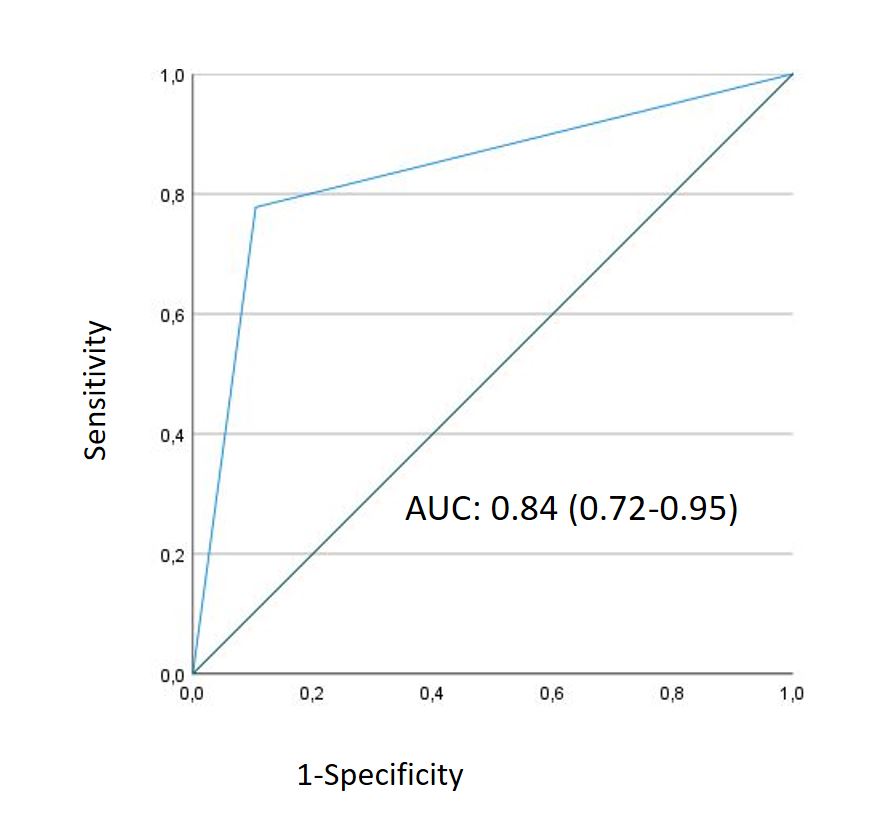


Figure S: Receiver operating characteristic (ROC) curves of diagnostic accuracy of Photon-Counting Detector CTA compared to DSA.
