# Supplementary material for: Photon-Counting CT-Angiography to Assess Intracranial Stents and Flow Diverters in Comparison to Digital Subtraction Angiography
Source: Clin Neuroradiol. 2025 May 9;35(4):669–77. doi: 10.1007/s00062-025-01519-2 (PMC12552290; doi:10.1007/s00062-025-01519-2)
Supplement: Supplementary file 2 — Table S1–S3 [file 62_2025_1519_MOESM2_ESM.docx]

Table S1 Reconstructions: Quantitative image quality measurements of the PCD-CT.

|  | Iodine | Pure lumen | UHR | VMI | P |
| --- | --- | --- | --- | --- | --- |
| Lumen of proximal vessel | | | | | |
| SNR | 9.2 ± 2.3 | 16.7 (29.8)* | 6.7 (4.3) * | 17.2 (7.5) * | <.001 |
| CNR | 233.6 (84) * | 27.12 (15.8)* | 9.1 (4.7) * | 21.9 ± 7.8 | <.001 |
| Lumen of proximal stent/flow diverter | | | | | |
| SNR | 7,4 ± 3.7 | -8.8 (19,1)* | 3.6 (5.6) * | 9.6 (6.4) * | <.001 |
| CNR | *223 (60) | 1.5 (10.3) * | 11.2 ± 6.3 | 24.7 ± 8.9 | <.001 |
| Lumen of stent/flow diverter | | | | | |
| SNR | 8.4 ± 5.5 | 3.6 ± 5.3 | 6.6 ± 5.5 | 16.6 ± 10.5 | <.001 |
| CNR | 219.5 (93) * | 12.4 (18.9)* | 10.0 (4.8)* | 21.3 (12) * | <.001 |

All values are reported are means ± SD or medians (IQR). Medians are marked with a *.

(*. Iod = iodine, PL = pure lumen, UHR = Ultra high-resolution polyenergetic reconstructions, VMI= Virtual monoenergetic reconstructions. (CNR = Contrast-to-noise Ratio, IQR: Interquartile range, SD: standard deviation, SNR: signal- to-noise ratio)

Table S2 Pairwise comparison of spectral and UHR reconstructions at three locations (parent vessel, proximal stent (stent markers), Stent lumen.

|  | Proximal vessel | | Proximal stent | | Stent | |
| --- | --- | --- | --- | --- | --- | --- |
|  | SNR | CNR | SNR | CNR | SNR | CNR |
| PL- UHR | <.001 | <.001 | .79 | .99 | .357 | .27 |
| PL - Iod | .010 | .023 | .001 | <.001 | .142 | .001 |
| PL-VMI | .99 | .79 | <0.001 | .012 | <.001 | .99 |
| UHR-Iod | .99 | <0.001 | .17 | <.001 | .99 | <.001 |
| UHR-VMI | <.001 | .050 | .001 | .085 | .034 | .004 |
| IOD-VMI | .002 | <.001 | .61 | .085 | .10 | .072 |

P: p-value adapted after Bonferroni-Correction. (CNR: Contrast-to-noise Ratio, IQR: Interquartile range, SD: standard deviation, SNR: signal- to-noise ratio)

Table S3 SNR values at the defined three different vessel sites for patients with stent or flow diverter, for patients with a minimal in-stent-diameter of <0,2cm and over >0.2cm as well as patients with and without additional implanted device (coil/clip).

|  | Stent/Flowdiverter | | P | Minimal stent diameter | | P | Additional implant | | p |
| --- | --- | --- | --- | --- | --- | --- | --- | --- | --- |
|  | Stent | Flow diverter |  | <0.2cm | >0.2cm |  | Absent | present |  |
| Iod PV | 9.5 ±2.9 | 9.3 ± 1.8 | .497 | 9.5 ± 2.7 | 9.0 ± 1.4 | .683 | 9.3 ±2.4 | 9.3 ±2.3 | .793 |
| Iod PS | 5.7 ± 3.5 | 8.0 ±3.9 | .497 | 5.7 ±3.5 | 9.5 ± 2.9 | .022 | 7.3 ± 3.9 | 7.8 ±2.9 | .99 |
| Iod S | 8.7 ± 6.1 | 8.7 ± 5.6 | .842 | 8.7 ± 6.1 | 8.1 ± 4.8 | .99 | 9.8 ±4.8 | 1.4 ± .94 | .002 |
| PL PV | 10.6(31.0.)* | 17.6 (34.2)* | .99 | 10.6 (31)* | 18.2 (33.9)* | .027 | 20.3 ±16.3 | 40.0 () * | .138 |
| PL PS | .8 ±1.8 | 3.4 ±3.7 | .356 | .8 ±1.8 | 4.2±3.5 | .014 | 1.9 (5.1)* | 1.8 ±1.2 | .99 |
| PL S | 3.7 ± 6.5 | 3.1 ±3.8 | .40 | 3.7 ±6.5 | 3.4 ± 3.8 | .661 | 4.8 ± 4.7 | -2.5 ± 4.0 | .047 |
| UHR PV | 6.8 (2.5)* | 7.2 (5.9) * | .182 | 6.8 (2.5) * | 8.4 ± 4.1 | .87 | 7.6 (4.2)* | 5.7 ±1.1 | .254 |
| UHR PS | 1.6 (4.1) * | 5.7 ± 3.9 | .065 | 1.6 (4.1)* | 6.4 ± 3.6 | .041 | 11.6 (7.3) * | 4.6 ±3.5 | .99 |
| UHR S | 6.8 ±6.9 | 6.9 ± 2.9 | .549 | 6.7 ± 6.9 | 6.4 ± 3.7 | .661 | 6.1 (6.5)* | .18 ±5.2 | .014 |
| VMI PV | 15.2 ± 4.1 | 19.0 (11.1)* | .842 | 15.2 ± 4.1 | 19.5 (21) * | .327 | 16.3 (8.8) * | 20.3 ± 4.9 | .99 |
| VMIPS | 7.6 ± 4.6 | 26.9 ± 10.0 | .013 | 7.6 ± 13.0 | 10.0 ± 9.1* | .102 | 9.2 ±5.9 | 11.7 ±1.3 | .138 |
| VMI S | 14.3 ±10.3 | 18.5 ± 9.7 | .234 | 14.3 ± 10.3 | 19.4 ± 10.6 | .40 | 17.8 ± 10.5 | 10.4 ±9.8 | .559 |

P: p-value adapted after Bonferroni-Correction. All values are reported are means ± SD or medians (IQR). Medians are marked with a *.

Abbreviations: Iod = iodine, PL = pure lumen, PS = SNR in in-stent vessel at the proximal stent end, PV = SNR in the vessel immediately proximal to the stent, UHR = Ultra high-resolution polyenergetic reconstructions, S= SNR in in-stent vessel at the narrowest stent site VMI= Virtual monoenergetic reconstructions (VMI).

IQR: Interquartile range, SD: standard deviation, SNR: signal- to-noise ratio.

CNR

|  | Stent/Flowdiverter | | P | Minimal stent diameter | | P | Additional implant | | p |
| --- | --- | --- | --- | --- | --- | --- | --- | --- | --- |
|  | Stent | Flow diverter |  | <0.2mm | >0.2mm |  | Absent | Present |  |
| Iod PV | 189.2 ±64.8 | 220.8 ±43.1 | .604 | 189.2 ± 64.8 | 246.5 (7.1) * | .102 | 240 (91.0) * | 202.0 ±48.7 | .634 |
| Iod PS | 184.3 ±94.8 | 227.0 (55.5)* | .604 | 184 ±94.5 | 229.8 ± 42.8 | .567 | 226.0 (58.0)* | 199.7 ± 52.2 | .712 |
| Iod S | 214.5 (94.7)* | 219.1 ± 30.5 | .315 | 214.5 (94)* | 223.5 (97.5)* | .7 | 228.0 (69)* | 113 ± 115 | .109 |
| PL PV | 23.4 ±13.8 | 29.4 ±15.4 | .604 | 24.1 ± 13.8 | 33.9 ± 7.7 | .072 | 29 2 ±12.8 | 25.4 ± 10.9 | .303 |
| PL PS | -31.6 (77.9)* | 10.5 (34.3)* | .053 | -.6 (77.9) * | 12.11 (28)* | .009 | 8.4 (36.6) * | 6.2 ± 6.4 | .87 |
| PL S | 6.2 ± 26.2 | 17.6 (15.8)* | .661 | 6.3 ± 26.1 | 24.2 (13.1)* | .24 | 23.6 (15.3) * | -13.2 ±14.6 | .014 |
| UHR PV | 7.0 (3.2) * | 11.1 ±4.1 | .065 | 7.9 (3.2)* | 11.1 ± 4.5 | .141 | 8.8 (6.0)* | 8.4 ±1.9 | .875 |
| UHR PS | 9.7 ±7.4 | 11.3 ± 6.1 | .99 | 9.7 ± 7.4 | 13.1 ± 4.2 | .374 | 11.8 ±6.3 | 8.4 ± 6.4 | .65 |
| UHR S | 10.5 (5.6) * | 10.1 ±3.7 | .968 | 10.5 (5.6)* | 10.8 ± 4.3 | .97 | 10.6 ± 3.5 | -7.6 ± 32.8 | .634 |
| VMI PV | 19.2 ± 5.7 | 23.83 ± 9.0 | .079 | 19.2 ±5.7 | 20.6 (15.4)* | .121 | 22.2 ±8.4 | 17.6 ()* | .99 |
| VMIPS | 22.5 ± 6.8 | 26.9 ±10.0 | .156 | 22.5 ±6.8 | 27.5 ± 10.8 | .513 | 22.2 (14.9) * | 16.9 ± 8.6 | .254 |
| VMI S | 20.4 (8.3)* | 26.6 ± 9.6 | .243 | 19.6 (8.3) * | 27.6 ± 9.9 | .40 | 21.7 (14.4)* | 14.4 ± 19 | .559 |

P: p-value adapted after Bonferroni-Correction. All values are reported are means ± SD or medians (IQR). Medians are marked with a *.

Abbreviations: Iod = iodine, PL = pure lumen, PS = SNR in in-stent vessel at the proximal stent end, PV = SNR in the vessel immediately proximal to the stent, UHR = Ultra high-resolution polyenergetic reconstructions, S= SNR in in-stent vessel at the narrowest stent site VMI= Virtual monoenergetic reconstructions (VMI).

IQR: Interquartile range, SD: standard deviation, CNR: Contrast- to-noise ratio.
