# Supplementary material for: Photon-Counting CT-Angiography to Assess Intracranial Stents and Flow Diverters in Comparison to Digital Subtraction Angiography
Source: Clin Neuroradiol. 2025 May 9;35(4):669–77. doi: 10.1007/s00062-025-01519-2 (PMC12552290; doi:10.1007/s00062-025-01519-2)
Supplement: Supplementary file 3 — Scanning protocol [file 62_2025_1519_MOESM3_ESM.docx]

**Scanning protocol**

Patients underwent a CT-Angiography (CTA) centered on the implanted device acquired on a clinical first-generation PCD-CT scanner (NAEOTOM Alpha, Siemens Healthineers, Erlangen, Germany) operated in ultra-high-resolution (UHR) mode, resulting in a reconstructed slice thickness of 0.2 mm and slice increment of 0.1mm. Spectral reconstructions were reconstructed in 0.4 mm slice thickness and 0.2 mm slice increment. The reconstructed matrix size was 1024 x 1024, and the field of view was adjusted for each patient to optimally image the stented vessels. For PURE LUMEN (PL)-reconstructions only 512 x 512 matrix size was available. The following acquisition parameters were used: tube voltage 140 or 120kvp. CTA was performed after the administration of 80 ml iodinated contrast material (Ultravist-370 (generic name, iopromide; Bayer Healthcare, Berlin, Germany), injected through a 20-gauge intravenous antecubital vein catheter using a power injector. The flow rate 4ml/s. Opacification of the common carotid artery was monitored using a bolus tracking technique. The start time of data acquisition was determined with a fixed delay of five seconds after the attenuation threshold was reached. Iterative reconstruction (denoted “QIR” by the manufacturer) level 3 was used for all PCD-CTA images. Polyenergetic reconstructions in UHR-mode as well spectral reconstructions were performed for Iodine, Pure Lumen, Virtual monoenergetic images (VMI). Images were reconstructed using the Bv56 and Bv72 kernel type. For PL- and Virtual monoenergetic Images-Reconstructions one keV levels (40) was reconstructed for each kernel
